# A New Organellar Complex in Rat Sympathetic Neurons

**DOI:** 10.1371/journal.pone.0010872

**Published:** 2010-05-27

**Authors:** Matt S. Ramer, Mario A. Cruz Cabrera, Nima Alan, Angela L. M. Scott, Jessica A. Inskip

**Affiliations:** International Collaboration on Repair Discoveries (ICORD), the University of British Columbia, Vancouver, British Columbia, Canada; Universität Heidelberg, Germany

## Abstract

Membranous compartments of neurons such as axons, dendrites and modified primary cilia are defining features of neuronal phenotype. This is unlike organelles deep to the plasma membrane, which are for the most part generic and not related directly to morphological, neurochemical or functional specializations. However, here we use multi-label immunohistochemistry combined with confocal and electron microscopy to identify a very large (∼6 microns in diameter), entirely intracellular neuronal organelle which occurs singly in a ubiquitous but neurochemically distinct and morphologically simple subset of sympathetic ganglion neurons. Although usually toroidal, it also occurs as twists or rods depending on its intracellular position: tori are most often perinuclear whereas rods are often found in axons. These ‘loukoumasomes’ (doughnut-like bodies) bind a monoclonal antibody raised against beta-III-tubulin (SDL.3D10), although their inability to bind other beta-III-tubulin monoclonal antibodies indicate that the responsible antigen is not known. Position-morphology relationships within neurons and their expression of non-muscle heavy chain myosin suggest a dynamic structure. They associate with nematosomes, enigmatic nucleolus-like organelles present in many neural and non-neural tissues, which we now show to be composed of filamentous actin. Loukoumasomes also separately interact with mother centrioles forming the basal body of primary cilia. They express gamma tubulin, a microtubule nucleator which localizes to non-neuronal centrosomes, and cenexin, a mother centriole-associated protein required for ciliogenesis. These data reveal a hitherto undescribed organelle, and depict it as an intracellular transport machine, shuttling material between the primary cilium, the nematosome, and the axon.

## Introduction

Organelles are subcellular compartments or macromolecular complexes with distinct structures and functions [1]. As some of the most architecturally-complex cells, neurons contain some highly-specialized organelles. An obvious example is the photon-detecting modified primary cilium of retinal photoreceptors. Another neuron-specific membranous organelle is the dendritic lamellar body, putatively related to dendrodendritic gap junctions in the olive [2]. One of several organelles lacking a limiting membrane is the nematosome, a nucleolus-like cytoplasmic inclusion of unknown composition and function found in all rat noradrenergic sympathetic ganglion neurons [3], and in many other neural and embryonic tissues of various species [4]. Other nemastosome-like inclusions (botrysomes or stigmoid bodies) contain proteins associated with synaptic plasticity and neurite outgrowth [5], [6], [7].

A serendipitous observation led us to another, surprisingly large (as large as rat erythrocytes and second only to the nucleus as a discrete intracellular structure) non-membranous toroidal organelle in sympathetic ganglion neurons. We call this structure the ‘loukoumasome’ from the Greek *loukoumas* (doughnut) and *soma* (body). We report on its composition, its distribution amongst sympathetic ganglia and amongst subclasses of sympathetic neurons, and on its subcellular localization and relationship with other organelles. It expresses non-muscle heavy chain myosin and centrosome-associated proteins, but is not itself a modified centrosome. It is found throughout the sympathetic chain, but exclusively within neurons expressing neuropeptide Y and calbindin-D28k. It is found throughout the cell body cytoplasm, as well as within the initial axon segment where it is linear rather than toroidal. Intriguingly, the loukoumasome associates with the nematosome, and with the centrosome and its primary cilium. These characteristics call to mind a dynamic organelle travelling non-randomly between cytoplasmic compartments, possibly facilitating communication between them.

## Results

### Morphology, distribution among ganglia and cytoskeletal antigenicity

**Figure 1:**
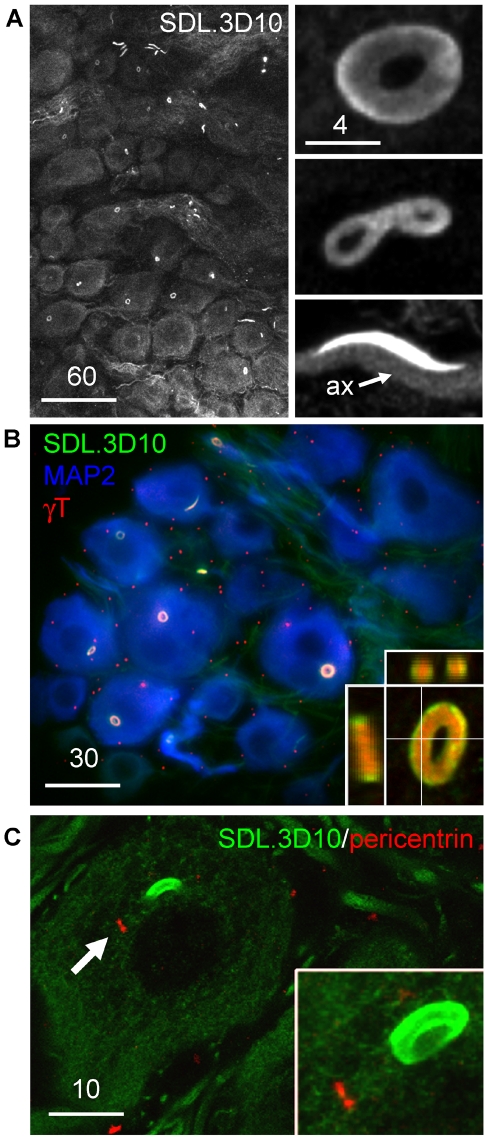
The loukoumasome. **A,** Loukoumasomes and their morphological variants in whole-mounted pelvic ganglion (confocal stack). ax = axon. **B**, The loukoumasome contains γ-tubulin (γT). Glial centrosomes are apparent as small dots (pelvic ganglion, standard epifluorescence, z-stack, maximum projection). Inset: confocal orthogonal view showing distribution of SDL.3D10 and γ-tubulin immunoreactivity. MAP2: microtubule-associated protein 2. **C**, Unlike centrioles (arrow, single confocal slice), loukoumasomes are pericentrin-negative (Inset: confocal stack). Scale bar units: µm.

A monoclonal antibody raised against neuron-specific βIII-tubulin (clone SDL.3D10 [8]) revealed a large (6.02±0.07 µm diameter, n = 222) intensely-staining perinuclear toroidal structure (the loukoumasome) occurring singly in adult rat sympathetic ganglia (Fig. 1A). A survey of the central and peripheral nervous systems indicated that these organelles are unique to peripheral autonomic ganglia, including pelvic, hypogastric, lumbar sympathetic, mesenteric, stellate and superior cervical ganglia, as well as in neurons embedded within the adrenal medulla. They were most abundant in pelvic and stellate ganglia. Less frequently-occurring morphological variants included linearized loukoumasomes and twisted or pinched figure-of-eight shapes (Fig. 1A, right panels). The morphological distribution was 54% tori, 30% linear, and 16% twisted/pinched tori (n = 184 from four rats). Toroidal diameter varied weakly with neuronal profile diameter (Pearson Product Moment correlation: R = 0.43, P<0.001, n = 222). They were present in male and female rats of three strains (Wistar, Sprague-Dawley and Long-Evans) from different sources (UBC Animal Care Centre and Charles River Laboratories). They were also apparent, though more difficult to find given their small size, in seven day old rat pups (data not shown). Confocal microscopy revealed that all three morphological variants were ‘hollow’ with respect to SDL.3D10 immunohistochemistry (Fig. 1B, inset).

Since loukoumasomes occur singly within neurons, bind the SDL.3D10 monoclonal antibody, and are in their toroidal form perinuclear, we asked whether they shared other characteristics with the centrosome. The loukoumasome's interior was intensely immunoreactive for the microtubule nucleator γ-tubulin (like most centrosomes but unlike those in mature neurons [9]) (Fig. 1B). Pericentrin immunoreactivity revealed obvious neuronal and glial centrioles, but did not label the loukoumasome (Fig. 1C). Loukoumasomes stained less intensely than the surrounding cytoplasm for γ-tubulin and medium-weight neurofilament (Fig. S1).

Surprisingly, loukoumasomes did not bind TuJ1, another widely-used βIII-tubulin antibody raised against a nearly-identical C-terminal isotype-defining epitope of the protein (Fig. S1), nor did they bind monoclonal antibodies specific for either polymerized (SMI-62) or depolymerized β-tubulin (SMI-61) (Fig. S1), suggesting that the antibody detects something other than βIII tubulin. Despite this, Western blots from pelvic ganglion showed single bands of the expected size using both the TuJ1 and SDL.3D10 antibodies (Fig. S1).

### Distribution among neurons

**Figure 2:**
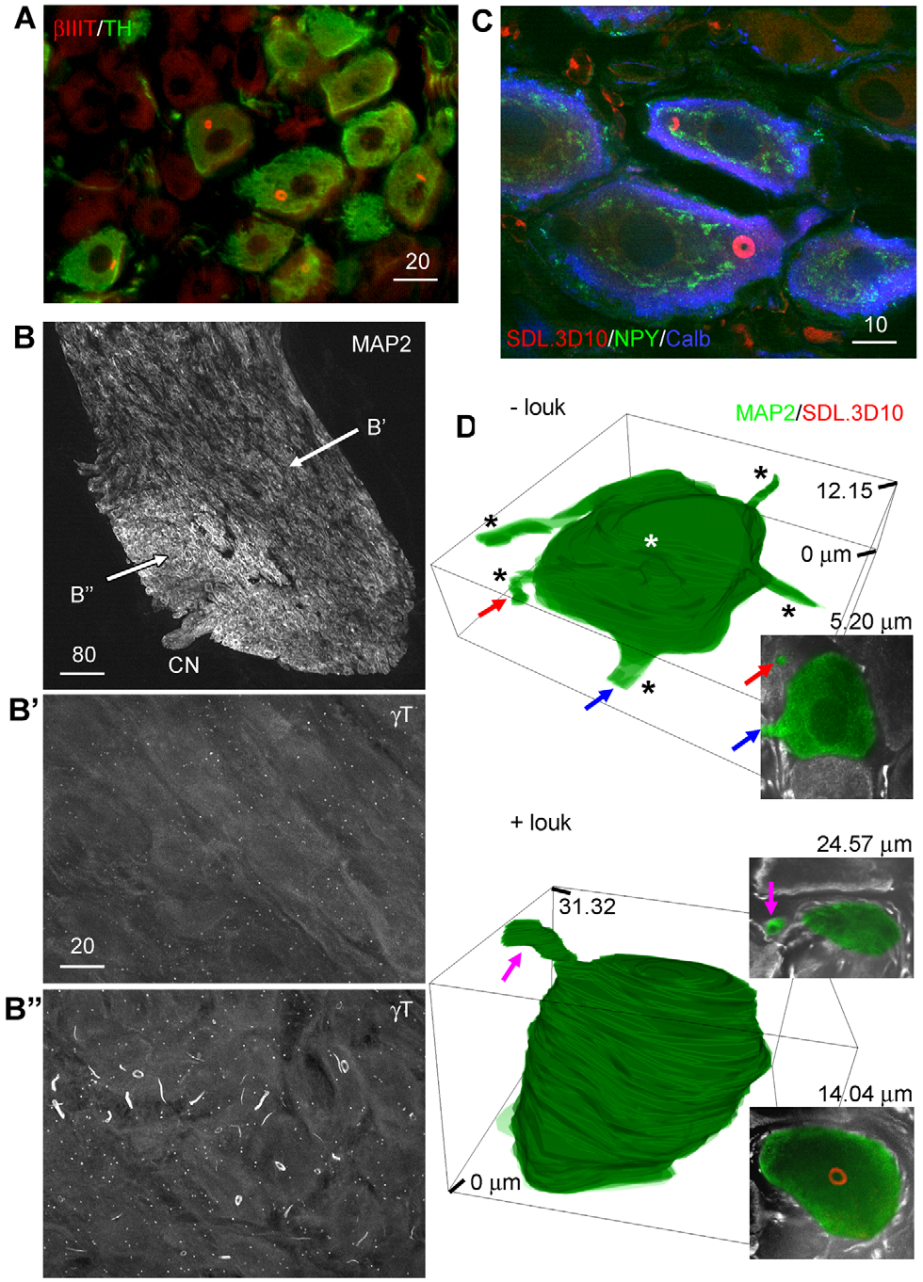
Distribution of the loukoumasome among neurons. **A**, Loukoumasome-containing neurons are TH-positive (pelvic ganglion, standard epifluorescence). **B**, In the stellate ganglion, loukoumasome-containing neurons are larger, more densely packed, and situated near the exit point of the cardiac nerve (CN). MAP2: microtubule-associated protein 2. **B′** and **B″** indicate the regions enlarged in the lower panels (confocal stacks, maximum projections). **C**, Loukoumasomes occur exclusively in a subset of neurons expressing both neuropeptide Y (NPY) and calbindin-d28k (Calb) (stellate ganglion, single confocal slice). **D**, Neurons with loukoumasomes (+louk) in the stellate ganglion have fewer processes than those without (-louk). Insets: confocal slices at the depths indicated. Coloured arrows indicate the same processes in confocal slices and 3D reconstructions. See also Videos S1 and S2. Scale bar units: µm.

Neurons which contained loukoumasomes were strictly adrenergic. This was particularly apparent in the mixed adrenergic/cholinergic male pelvic ganglion (Fig. 2A). Although loukoumasomes were relatively uniformly-distributed among throughout pelvic ganglia (among noradrenergic neurons), loukoumasome-containing neurons often occurred in a distinct region within the stellate ganglion close to the exit point of the cardiac nerve. These cells were larger, rounder and more densely packed than those in loukoumasome-negative regions of the ganglia (Fig. 2B), and we found that we could reliably predict the location of loukoumasomes based on these morphological characteristics. The size, shape and intraganglionic position (Fig. 2B) of loukoumasome-positive stellate ganglion neurons correspond precisely with a subpopulation of large round neurons innervating the left ventricle of the heart [10], [11].

In both stellate and pelvic ganglia, a subset of large, round neurons co-express neuropeptide Y (NPY) and calbindin-D28k [11], [12]. Several previous findings strongly suggest that this particular sympathetic neurochemical phenotype represents the population of neurons which contains loukoumasomes. First, the location and shape of loukoumasome-containing neurons in the stellate ganglion is identical to those which express both calbindin and NPY [11]. Second, in pelvic ganglia, where all TH-positive neurons contain NPY [13], approximately half (48–51.1%) of the TH-positive neurons also express calbindin [12], corresponding very well with the proportion of noradrenergic neurons containing loukoumasomes (59±7%). Third, all superior cervical ganglion neurons projecting to the pineal gland express both NPY and calbindin [14], and occur with a frequency (1–2%) [15], roughly equivalent to what we found for noradrenergic neurons containing loukoumasomes (4±2%). Indeed, we found that, without exception, loukoumasome-containing neurons in the pelvic and stellate ganglia (100 neurons from each) expressed both NPY and calbindin (Fig. 2C).

In pelvic ganglia where nearly all noradrenergic cells contain loukoumasomes, neurons are unipolar [18]. We therefore asked whether those in stellate ganglia which contained loukoumasomes were also structurally simpler (as suggested by their roundness and dense packing) than those which did not. We counted the number of MAP2-positive processes emerging from neuronal somata completely contained within confocal stacks of 50 µm-thick longitudinal sections of loukoumasome-negative regions and loukoumasome-containing neurons within stellate ganglia (Fig. 2D). It should be noted that only processes emerging from the neurons which were parallel or near-parallel to the plane of section were counted as those emerging orthogonally were difficult to identify reliably. For loukoumasome-negative neurons, the mean maximum z-size was 11.28±0.8 µm (n = 29), and 17.21±0.95 µm (n = 25) for those containing loukoumasomes (P<0.0001, Student's t-test). Because of the plane of section (parallel to the broad side of the ganglion), these distances in the z-plane are smaller than the mean diameter in the xy plane (23.0±0.2 µm for loukoumasome-containing neurons). The mean number of primary processes (emerging near-parallel to the plane of section) was 3.45±0.20 and 1.12±0.13 for loukoumasome-negative and loukoumasome-positive neurons, respectively (P<0.0001, Student's t-test). Serial reconstructions of loukoumasome-negative and loukoumasome-positive neuronal somata and primary processes (Fig. 2D, and see Videos S1 and S2) confirmed the relative structural simplicity of the latter.

### Subcellular distribution

**Figure 3:**
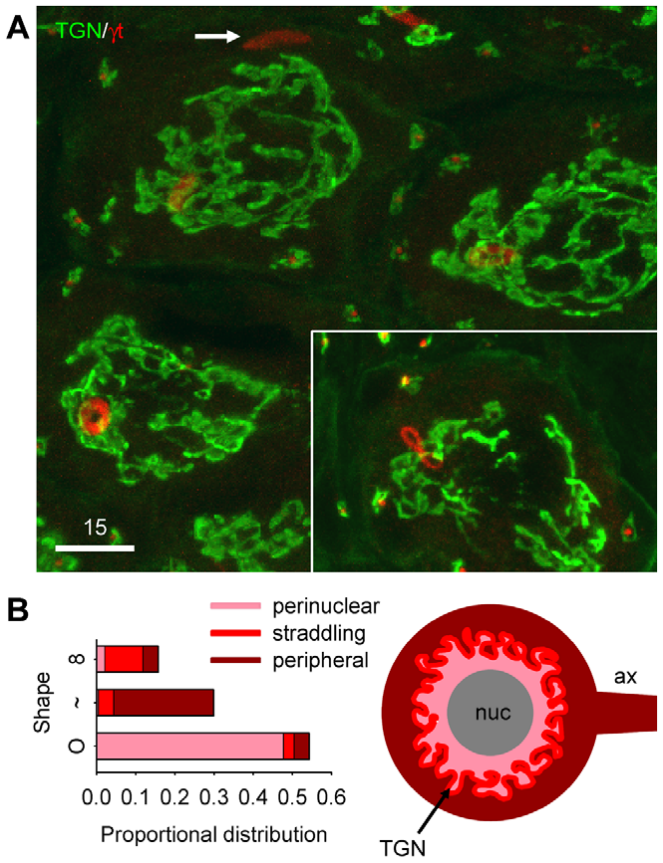
Subcellular distribution of the loukoumasome. **A**,**B**, Relationship to the trans-Golgi network (TGN), which defines perinuclear and sub-cortical compartments. Tori occur primarily in the perinuclear compartment, linearized variants more peripherally and often in the axon (arrow in **A**), and figure-eights straddle the TGN. γT: γ-tubulin. Pelvic ganglion, confocal stack. See also Videos S3 and S4. Scale bar units: µm.

**Figure 4:**
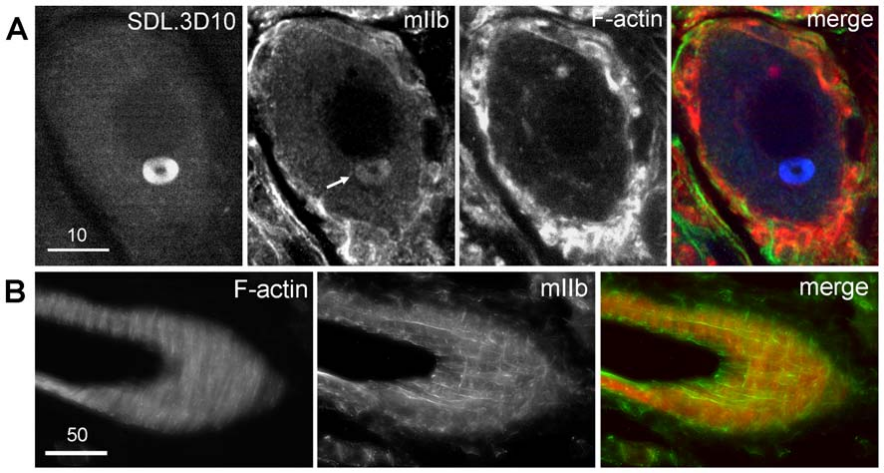
Loukoumasomes contain non-muscle heavy-chain myosin (mIIb). mIIb co-localizes with sub-plasmalemmal F-actin, as expected, but also to the F-actin-negative loukoumasome (arrow). Stellate ganglion, single confocal slice. Scale bar units: µm.

To better define the subcellular distribution of loukoumasomes, we related their position (in confocal stacks) to the trans-Golgi network (TGN) which in neurons demarcates the perinuclear compartment (Fig. 3). As tori, loukoumasomes were nearly always on the nuclear side of the TGN (88/100); in their linear form, they were usually peripheral to the TGN (47/55), most often in axons (axonal versus dendrite localization was confirmed in the pelvic ganglion, where neurons are unipolar [16] (Fig. 1A, inset)). In the majority of cases (18/29), twisted/pinched loukoumasomes straddled the TGN (see also Videos S3 and S4). These results suggest that the loukoumasome is a dynamic structure, shuttling between the perinuclear compartment and the axon. Further suggesting motility, the loukoumasome bound an antibody specific for non-muscle heavy chain myosin (clone 3H2) (Fig. 4). However, segments of the large pelvic nerve (efferent limb) and cavernous nerves taken 1–2 mm away from the pelvic ganglion revealed no loukoumasomes. We also asked whether loukoumasomes accumulated at a ligature placed 12 hours earlier around the same nerves. Results from these experiments were negative, suggesting that the organelles, if indeed motile, travel only as far as the initial axonal segment. Longer survival times (48 hours) following crush of the same nerves resulted in a significant reduction in the number of loukoumasomes from the ganglion, from 2991±554/mm^3^ to 442±244/mm^3^ (Student's t-test: P = 0.004, n = 4). They also disappeared completely in overnight dissociated pelvic ganglion neuron cultures (data not shown).

**Figure 5:**
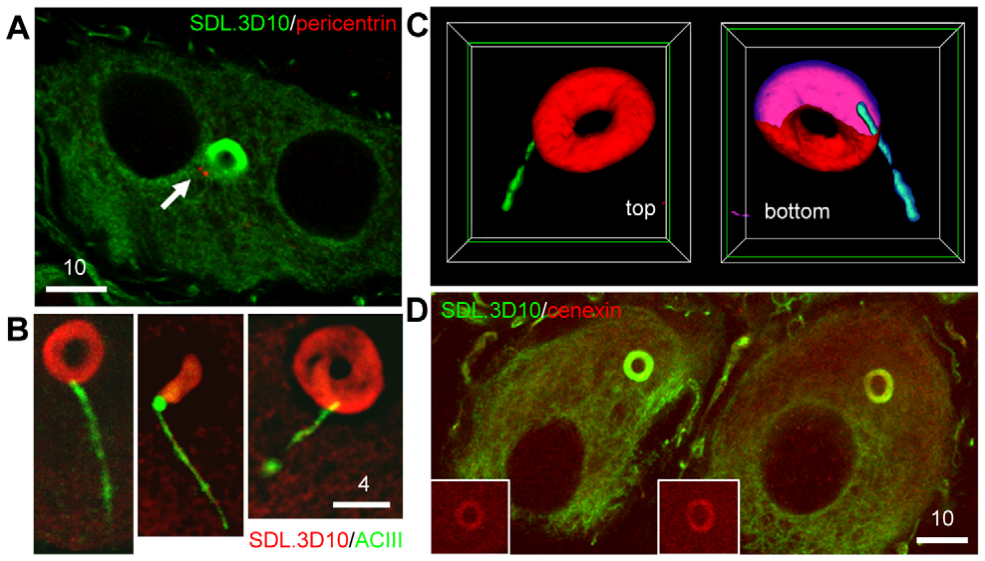
Loukoumasomes associate non-randomly with the primary cilium. **A**, Pericentrin-positive centrioles close to the loukoumasome in a binucleate pelvic ganglion neuron (single confocal slice). **B**, Examples of ‘ciliated’ loukoumasomes (stellate ganglion, single confocal slices). ACIII: adenylyl cyclase III. **C**, surface-rendered, cutaway reconstruction of a loukoumasome with an embedded primary cilium. Interior surfaces are coloured magenta (loukoumasome) and cyan (primary cilium). Plane of slice indicated by green borders. See also Video S5. **D**, The loukoumasome contains cenexin, a mother centriole-related protein. Scale bar units: µm.

In differentiated and interphase cells, motile vesicles and regulatory complexes use the centrosome as a docking station [17], and so we asked if the loukoumasome showed any particular association with neuronal centrosomes. Pericentrin-immunoreactive centrioles were sometimes close to (Fig. 5A) and occasionally embedded within the loukoumasome. To investigate this apparent association further, we used adenylyl cyclase III (ACIII) immunoreactivity to identify the neuronal primary cilium, the basal body of which is formed by the mother centriole [18], [19]. Dual immunohistochemistry using SDL.3D10 and ACIII antibodies showed that 9.7% (16/165) of loukoumasomes contacted primary cilia. For 222 loukoumasome-containing neurons, we calculated the toroidal volume as a fraction of the neuronal volume (less that occupied by the nucleus), and found that the loukoumasome occupies 0.27±0.01% of the cytoplasm. This means that the association of loukoumasomes with primary cilia (and by extension, the mother centriole whose volume we can consider negligible), far exceeds that which would occur by chance (∼0.5/165, or 1/330, P<0.001, z-test). We also asked if the loukoumasome contains another mother centriole-related protein, cenexin [20]. In control tissue (rat epididymal sperm), the antibody labelled caudal to the middle-piece/principal piece boundary of the sperm tail (data not shown), as has been described previously [21]. The loukoumasome also stained positively for cenexin (Fig. 5D). These results strongly suggest that the centrosome also serves as an intracellular target of the loukoumasome, and further that it may contribute to ciliogenesis or ciliary maintenance.

### Loukoumasomes associate with nematosomes

**Figure 6:**
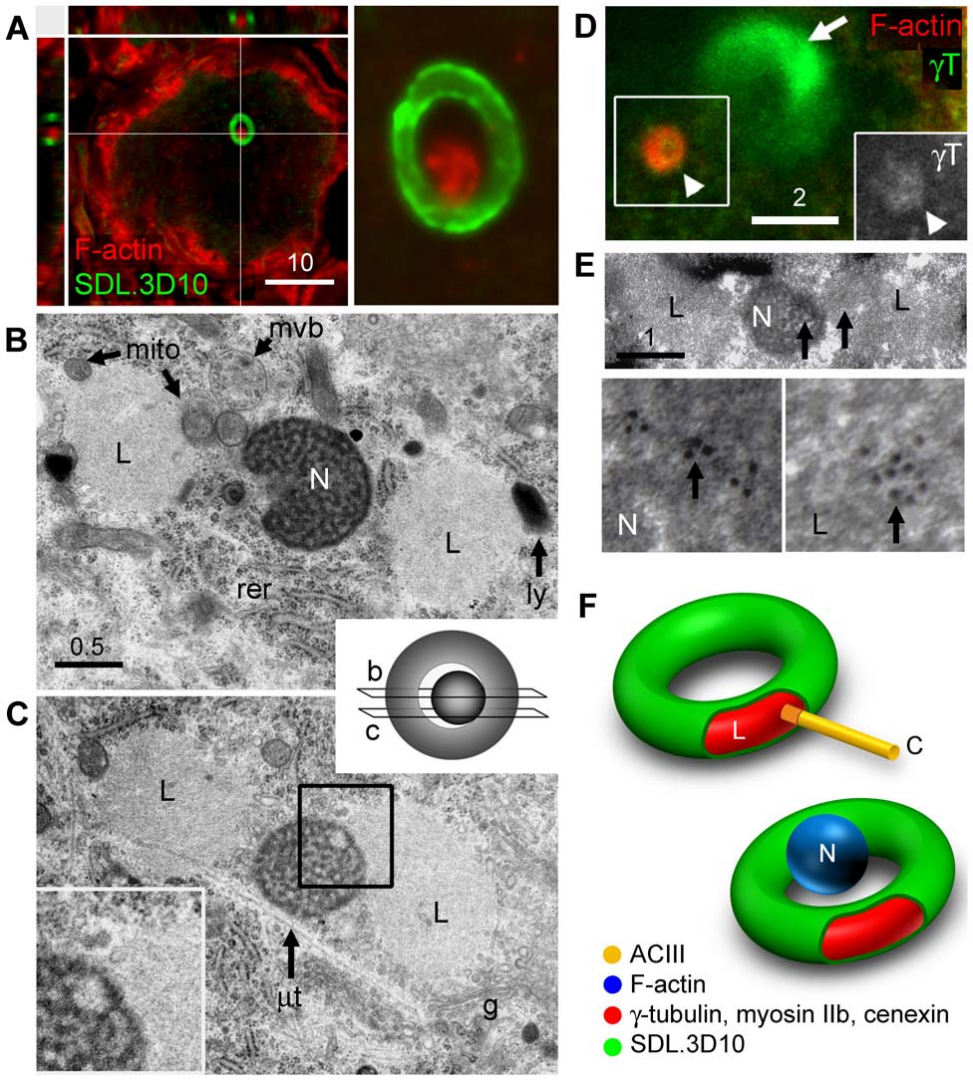
Loukoumasomes associate non-randomly with nematosomes. **A**, The loukoumasomal hole is occupied by an F-actin sphere. Left panel: confocal stack plus orthogonal views. Right panel, high-power view (single confocal slice). **B**, **C**, Loukoumasomal ultrastructure. N: nematosome; L: loukoumasome; mvb: multivesicular body; ly: lysosome; rer: rough endoplasmic reticulum; g: Golgi apparatus. Schematic: origins of sections shown in **B** and **C**. **C**, Contact between the nematosome and the loukoumasome (region indicated enlarged in inset). Note the similarity between the electron-lucent material within the nematosome and the loukoumasome. µt: microtubule. **D**, Loukoumasome-associated nematosomes are γ-tubulin-positive (γT) (single confocal slice). **E**, Immunoelectron microscopy confirming γT-immunoreactivity of the loukoumasome-nematosome complex (arrows: 10 nm gold particles). **F**, Schematic illustrating the composition of the loukoumasome/cilium and loukoumasome/nematosome complexes. L: loukoumasome; C: primary cilium; N: nematosome. Scale bar units: µm.

While phalloidin labelling of filamentous actin (F-actin) did not label loukoumasomes, it did reveal a roughly spherical perinuclear structure, ∼1 µm in diameter (Figs. 4A and 6A). This smaller organelle, which was present in more neurons than were loukoumasomes (all adrenergic and a few cholinergic neurons), most often had 1–3 pericentral F-actin-negative cavities, and occurred usually once, but occasionally twice or thrice, per neuron. For 37.8% of toroidal loukoumasomes (n = 111), their central holes were occupied by the F-actin-positive sphere. In these cases the sphere usually made contact with the inner loukoumasomal border (Fig. 6A). Loukoumasomes occupied by F-actin spheres did not have attached primary cilia. To determine the probability of spheres making contact with the loukoumasome by chance, we estimated the proportional volume of cytoplasm occupied by a 1 micron-thick layer coating the outside of the loukoumasomal torus. This calculation revealed that the contact would occur by chance with a frequency of 1.88±0.01%, significantly different from that observed (P<0.001, z-test). Like the centrosome, this sphere of F-actin is also a likely target of the loukoumasome.

Ultrastructurally (Fig. 6B, C), the loukoumasome was composed of relatively homogenous, electron-lucent granulofibrous material oriented more-or-less axially along the torus like thread on a bobbin. Unusually for an organelle of this size, it lacked a membrane. In accordance with the lack of SMI-62 immunoreactivity, the loukoumasome was not associated especially with microtubules. In the example shown, the loukoumasome's central hole was occupied by a nematosome. The nematosome, which occurs primarily in embryonic and neural tissues of several species [3], [4], [22], [23], bears many similarities to the F-actin sphere, including size, shape, internal structure, and frequency of occurrence. The lower inset in Figure 5C shows the two structures making direct contact. Moreover, the appearance of the more electron-lucent material within the nematosome is identical to that of the loukoumasome, suggesting a similar composition. High-power confocal images of the phalloidin-positive structure showed that it stained weakly for γ tubulin (Fig. 6D), but only when in the vicinity of a loukoumasome. Post-embedding immunoelectron microscopy confirmed that both the loukoumasome and associated nematosome were γ tubulin-positive (Fig. 6E). It therefore seems likely that the F-actin sphere and the nematosome are one and the same.

### A site of protein storage?

Although some have suggested that the nematosome is an RNA storage site [3], [22], [23], we found that neither the nematosome nor the loukoumasome bound the RNA-specific dye SYTO 14 (Fig. S2). Both structures were also negative for TCP-1γ, a chaperonin protein required for co-translational folding of actin, γ, β and γ-tubulins [24], [25] (Fig. S2). The nematosome/loukoumasome complex is therefore not a site of local cytoskeletal protein synthesis.

## Discussion

Given that the rat is arguably the best-studied vertebrate animal model, particularly with respect to the biology of sympathetic neurons, it is surprising that such a large organelle has gone unnoticed. The SDL.3D10 monoclonal antibody, raised against a synthetic peptide corresponding to the C-terminal end of human βIII tubulin (ESESQGPK, [8]) is commercially available from Sigma-Aldrich (catalogue number T8660), and widely-used as a pan-neuronal marker. It is perhaps because of the disappearance of the organelle with axotomy and in dissociated culture, or because most studies on sympathetic ganglion neurons use those from superior cervical ganglia (where loukoumasomes are rare), that it has been overlooked. Ultrastructural studies have most likely failed to reveal the loukoumasome because it occurs only once per neuron, and because, having an electron density comparable to that of the surrounding cytoplasm, it fails to catch the eye. In a search of the ultrastructural literature on sympathetic neurons, we have found only one example of a loukoumasome, which incidentally, was in close proximity to a nematosome [26]. Only the latter was commented upon, however.

Fibrillary inclusions which bear some ultrastructural resemblance to loukoumasomes, have been described in rat brain [27], although the fibres are thicker and the inclusions are bounded by a filamentous wall. ‘Granulofilamentous bodies’ in the rat thalamus are composed of fibres more closely-resembling those of loukoumasomes, and they also lack any sort of wall or membrane (http://synapses.clm.utexas.edu/atlas/contents.stm). An organelle with the closest ultrastructural resemblance to the loukoumasome has been described in the dragonfly oocyte (the ‘dense mass’ or ‘yolk nucleus’) [28], [22], [23]. Intriguingly, during oogenesis it forms together with the nematosome as diffuse perinuclear material which coalesces into the two structures.

What all of these filamentous inclusions lack in common with the loukoumasome is, of course, their shape. We know of only two other organelles which take the shape of a torus. One, from the plant *Arabidopsis thaliana*, is a highly motile organelle which transforms between toroidal and tubular morphologies via a figure-of-eight-like intermediate [29]. The other is the border of the ring canal, an intercellular cytoplasmic bridge connecting germline cells which has been most intensively studied in *Drosophila*. This actin-rich structure, which varies in size from 0.5 µm to 10 µm in diameter, forms as a result of incomplete cytokinesis [30]. This may give some clue as to the origin of the loukoumasome in sympathetic neurons, a proportion of which are binucleate and tetraploid (See Fig. 5 and [31]), meaning that the final karyokinesis is often not accompanied by cytokinesis. Could the loukoumasome be a remnant of the contractile ring left over from the cell's proliferative phase? Its expression of myosin II, the principal actuator of cytokinesis [32], [33], is certainly suggestive. The fact that the loukoumasome does not bind phalloidin means that it would have to have rid itself of (or never have incorporated) the F-actin comprising the contractile ring scaffolding. Thus it is perhaps not irrelevant that nearly 40% of loukoumasomes encircle F-actin-rich nematosomes. The expression of γ tubulin by the loukoumasome may also point to origins in the cytokinetic apparatus. In mitotic mammalian cells, γ tubulin localizes to the minus ends of microtubules forming the midbody [34], [35], a transient microtubular entity providing structural support for the cytoplasmic bridge connecting daughter cells during telophase.

Loukoumasomes are found in a neurochemically and structurally distinct subset of sympathetic ganglion neurons. Most, if not all of the defining features of separate sympathetic phenotypes are governed by peripheral targets of sympathetic axons [36], [37], [38]. This has been shown to be true for NPY and calbindin expression: neurons normally innervating the sublingual or submaxillary gland begin to express both markers if they instead innervate a transplanted pineal gland [39], [14]. Intriguingly, binucleate neurons are exclusively NPY/calbindin-positive (unpublished observations), raising the possibility that target innervation halts mitosis (sympathetic neuroblasts undergo mitosis while extending axons [40]). For some of these neurons, this would occur between nuclear division and cell division, resulting in the generation of a binucleate neuron, and perhaps also the loukoumasome. Intriguingly, calbindin itself, a calcium buffering protein, may actively participate in cytokinetic arrest, as calcium transients accompany, and indeed are required for cytokinesis [41]. Specific functions of calcium signals include recruitment of actin filaments to the contractile ring and regulation of actin-myosin interactions [42], [43], [44]. Although still speculative, the onset of calbindin expression may thus define the particular composition of the loukoumasomal torus – expressing myosin II but lacking F-actin.

In ascribing a function to the loukoumasome, we must be even more tentative. We are obliged to consider that it is no more than a developmental relic, but this is difficult to reconcile with the fact that such a large and undoubtedly costly structure grows with its parent neuron and is maintained into adulthood. Its inferred motility, and even more so its association with the centrosome and primary cilium also insinuate some utility. γ tubulin is lacking from the centrosomes of mature neurons [9], and although direct evidence for motility of the organelle is still lacking, it is possible that the loukoumasome periodically supplies the centrosome with this important microtubule nucleator in order to maintain cell shape. It may also contribute to the upkeep of the primary cilium (an important sensory organelle also once considered vestigial [45]), and along these lines it is intriguing that the loukoumasome also stains positively for cenexin, a protein localized to the mother centriole and required for ciliary development [20].

The strict definition of ‘organelle’ allows for the inclusion of phenotype-defining features of neurons such as axons, dendrites and in the case of the special senses, modified primary cilia. In all of these cases, the organelles in question are involved in transducing, transmitting and/or receiving physical or chemical cues, and thus bear an obvious relationship to the extracellular environment. The loukoumasome, by contrast, represents an entirely intracellular, phenotype-defining organelle. Although it is well-established that in mature neurons, structural, chemical and functional phenotype is governed by developmental lineage and extrinsic factors such as target-derived cues [46], [47], [48], [38], [49], these data also suggest the intriguing possibility that the loukoumasome, by maintaining the primary cilium or perhaps even by communicating ciliary signals to the cell's interior (including the axon), serves as a liaison between extrinsic information and cellular identity.

## Materials and Methods

### Animals

All experiments were approved by the University of British Columbia Animal Care Committee, and conformed to the guidelines of the Canadian Council on Animal Care. Adult Wistar (4 female, 26 male), Sprague-Dawley (4 female, 4 male) and Long-Evans rats (2 female, 2 male) were purchased from UBC animal care centre or Charles River Laboratories. Seven day-old Sprague-Dawley rat pups were purchased from the UBC animal care centre. Chloral hydrate-killed rats were perfusion-fixed with 4% paraformaldehyde. In isofluorane-anesthetized rats, the efferent pelvic nerve (sometimes referred to as the accessory nerve) and cavernous nerves were tightly ligated (n = 2) or transected (n = 4). Animals were killed 12 (n = 2) or 48 (n = 4) hours later. Most studies were carried out on pelvic and stellate ganglia, but other tissue examined included superior cervical, celiac, inferior mesenteric, dorsal root ganglia, adrenal medulla, midgut, spinal cord and brain.

### Immunohistochemistry and histochemistry

Tissue was sectioned at 20 µm on a cryostat and thaw-mounted onto glass slides and stored at -80C. For some experiments, 50 µm-thick longitudinal floating sections of sympathetic ganglia (four stellate ganglia from two rats) were collected in 0.1M phosphate buffer (PB), and mounted on glass slides following immunohistochemistry. In all cases, sections were blocked in 10% normal donkey serum in PBS (containing 0.2% Triton X-100, and 0.1% sodium azide) for 20 minutes. Primary antibodies were diluted in the same solution and included rabbit anti-γ tubulin (1∶1000, AbCam), rabbit anti-pericentrin (1∶1000, AbCam) mouse anti-βIII tubulin (clones: SDL.3D10, 1∶500, Sigma-Aldrich; TuJ1, 1∶1000, Covance), mouse anti-polymerized and de-polymerized β tubulin (clones SMI-61 and SMI-62, both 1∶1000, Covance), mouse anti-γ tubulin (clone B-5-1-2, 1∶1000, Sigma), mouse anti medium-weight neurofilament (NFM, 1∶1000, clone NF-09, AbCam), sheep anti-tyrosine hydroxylase (TH, 1∶100, Chemicon), mouse anti trans-Golgi network (clone 2F7.1, 1∶1000, Affinity Bioreagents), mouse anti non-muscle heavy-chain myosin (1∶2000, clone 3H2, Abcam), rabbit anti cenexin (1∶1000, Abcam), and rat anti TCP-1γ (1∶200, Stressgen). Secondary antibodies (1∶200) were conjugated to Alexa dyes 488, 546, or 647 (Invitrogen). Some sections were labelled with Alexa 546 or 647-conjugated phalloidin (1∶50), NeuroTrace (1∶1000), and the RNA-specific dye SYTO 14 (1∶1000, Invitrogen).

For epifluorescent imaging we used an Axioplan 2 fluorescent microscope (63x oil, 1.45 aperture) with a digital camera (Q Imaging) and Northern Eclipse software (Empix Imaging Inc.). Confocal imaging (63x and 100x oil-immersion objectives, apertures 1.4 and 1.45, respectively) employed a laser scanning microscope (Zeiss Pascal Excite on an upright AxioImager MI), and two spinning disk confocal microscopes (Quorum Wave FX, mounted on a Leica DMI6000 inverted microscope with a Hammamatsu CCD camera, and a Yokogawa Spinning disk on an inverted Zeiss AxioObserver Z.1 equipped with an AxioCam CCD camera).

### Quantification

Feret diameter (the diameter of a perfect circle with the same area as objects outlined) was measured for each torus and for the cellular profile containing it (SigmaScan Pro, SPSS). Correlations between profile diameter and toroidal loukoumasomal diameter were then carried out in SigmaStat (SPSS). To determine with what frequency neurons contained loukoumasomes, we counted the number of loukoumasomes in each of five fields per ganglion (superior cervical or pelvic) per animal. Counts were corrected using the Abercrombie correction factor [50]. This was followed by calculating feret diameter of all DβH-positive neurons in the same sections (since loukoumasomes are only in noradrenergic neurons). To convert the number of cellular profiles in each section to the equivalent number of neurons from which those profiles were derived, we employed recursive translation [51].

To detect changes in loukoumasomal and nematosomal number following axotomy, we counted the structures in pelvic ganglion regions comprising mostly of TH or DβH-immunoreactive neurons. We then calculated loukoumasomal number as a function of tissue volume.

In confocal images of 50 µm-thick sections of stellate ganglia stained for γ-tubulin and MAP2, we counted the number of primary processes of cells with and without loukoumasomes. To partially reconstruct neuronal somata and primary processes, we traced profiles in each confocal z-layer and reconstructed these as z-stacks using AxioVision software.

### Western Blotting

Pelvic ganglia of adult rats were removed, and the tissue was homogenized in 0.01 M Tris(hydroxymethyl)aminomethane (Tris) with protease inhibitors (Invitrogen, Carlsbad, CA). Homogenized samples of three different animals were combined in order to increase protein concentration, which was subsequently determined with BCA protein titration. Proteins were separated, 40 µg per lane, on 10% sodium dodecyl sulfate (SDS)–polyacrylamide gel electrophoresis and transferred to a PVDF transfer membrane (Amersham Corp., Arlington Heights, IL). The membranes were then blocked for 2 hours in 5% Bovine serum albumin (BSA, Sigma-Aldrich, St. Louis, MO), and incubated overnight with anti-βIII tubulin (TuJ1, 1∶5000, host mouse, Covance, Princeton, NJ). The membranes were then washed in 0.5M Tris, 0.15M NaCl, and 0.1% Tween 20, and incubated with goat anti-mouse IgG-horseradish peroxidase antibody (1∶5000, Santa Cruz Biotechnology Inc., Santa Cruz, CA) for 2 hours at room temperature. The membranes were washed again, treated for 5 minutes with enhanced chemiluminescence reagents (ECL; Amersham Corp.), and imaged with Biospectrum Biochemi500 Imaging System (Ultraviolet Products, Upland, CA). Subsequently, the membrane was stripped in 1M Tris, 20% SDS, distilled H_2_O and β-mercaptoethanol, and the same blotting procedure, as explained above, was repeated using another anti-βIII tubulin antibody (SDL.3D10, 1∶5000, host mouse) from Sigma-Aldrich.

### Electron and Immunoelectron Microscopy

Male rats were perfused through the heart with a 2.5% solution of EM-grade glutaraldehyde in sodium cacodylate buffer. Pelvic ganglia were immersion-fixed for a further hour, then rinsed in buffer. Whole ganglia were osmium-fixed (1%), washed in water (5 minutes), and dehydrated in ethanol (30%, 50%, 70%, 80%, 90%, 95%, 100%, 100%, 100%, in distilled water, 10 minutes each). Spurr's:Jembed resin (1∶1) was infiltrated in dry acetone (25%, 50%, 75%, 100%, 100%, 100%, one hour each), and polymerized overnight at 60°C.

Thin sections were cut using a Leica Ultracut T (Leica Microsystems AG, Wetzlar, Germany) on a 45° Diatome diamond knife. Sections were picked up on copper grids and stained in 2% uranyl acetate (12 min) and Reynolds' lead citrate (6 min). Sections were viewed in a Hitachi H7600 transmission electron microscope (Hitachi, Ltd, Tokyo, Japan) and photos were taken using a built-in AMT digital camera (American Technologies, Corp., San Francisco, CA).

For immuno-EM, rats were perfused with 4% PF and 0.1% glutaraldehyde, tissue was embedded in LR white, and primary antibody application (rabbit anti γ-tubulin) was followed by 10 nm gold-conjugated goat anti-rabbit secondary antibody.

## Supporting Information

Figure S1 Cytoskeletal antigenicity of the loukoumasome. A, The SDL.3D10 monoclonal antibody, but not the TuJ1 rabbit polyclonal, recognizes the loukoumasome. The two antibodies otherwise recognize identical structures. B, Loukoumasomes do not contain either polymerized (βT pol), or de-polymerized (βT depol) β-tubulins. γ-tubulin-positive (γT) loukoumasomes are weakly-positive for α tubulin subunits (αT) and medium-weight neurofilament (NFM). All images: single confocal slices. C, Western blot of pelvic ganglion tissue probed for TuJ1, stripped and re-probed for SDL.3D10. In both cases a single, prominent band is visible at ∼46 kDa. Scale bar units: μm.
(4.64 MB TIF)

Video S1 Loukoumasome-negative stellate ganglion neuron. Surface-rendered neuron obtained by tracing its profile in 50 micron-thick sections immunolabled for microtubule-associated protein 2 (MAP2).
(2.03 MB AVI)

Video S2 Loukoumasome-positive stellate ganglion neuron. Surface-rendered neuron obtained by tracing its profile in 50 micron-thick sections immunolabled for microtubule-associated protein 2 (MAP2).
(2.26 MB AVI)

Video S3 Toroidal loukoumasomes in the perinuclear compartment. Green: trans golgi network; red: gamma tubulin.
(1.19 MB MOV)

Video S4 Figure-of-eight loukoumasome straddling the trans-golgi network. Green: trans golgi network; red: gamma tubulin.
(15.28 MB AVI)

Video S5 Ciliated loukoumasome. Surface-rendered, cutaway reconstruction of a loukoumasome with an embedded primary cilium. Interior surfaces are coloured magenta (loukoumasome) and cyan (primary cilium). Plane of slice indicated by green borders.
(2.61 MB AVI)
