# Supplementary figures and images for: A New Organellar Complex in Rat Sympathetic Neurons
Source: PLoS One. 2010 May 27;5(5):e1000872. doi: 10.1371/journal.pone.0010872 (PMC2877718; doi:10.1371/journal.pone.0010872)

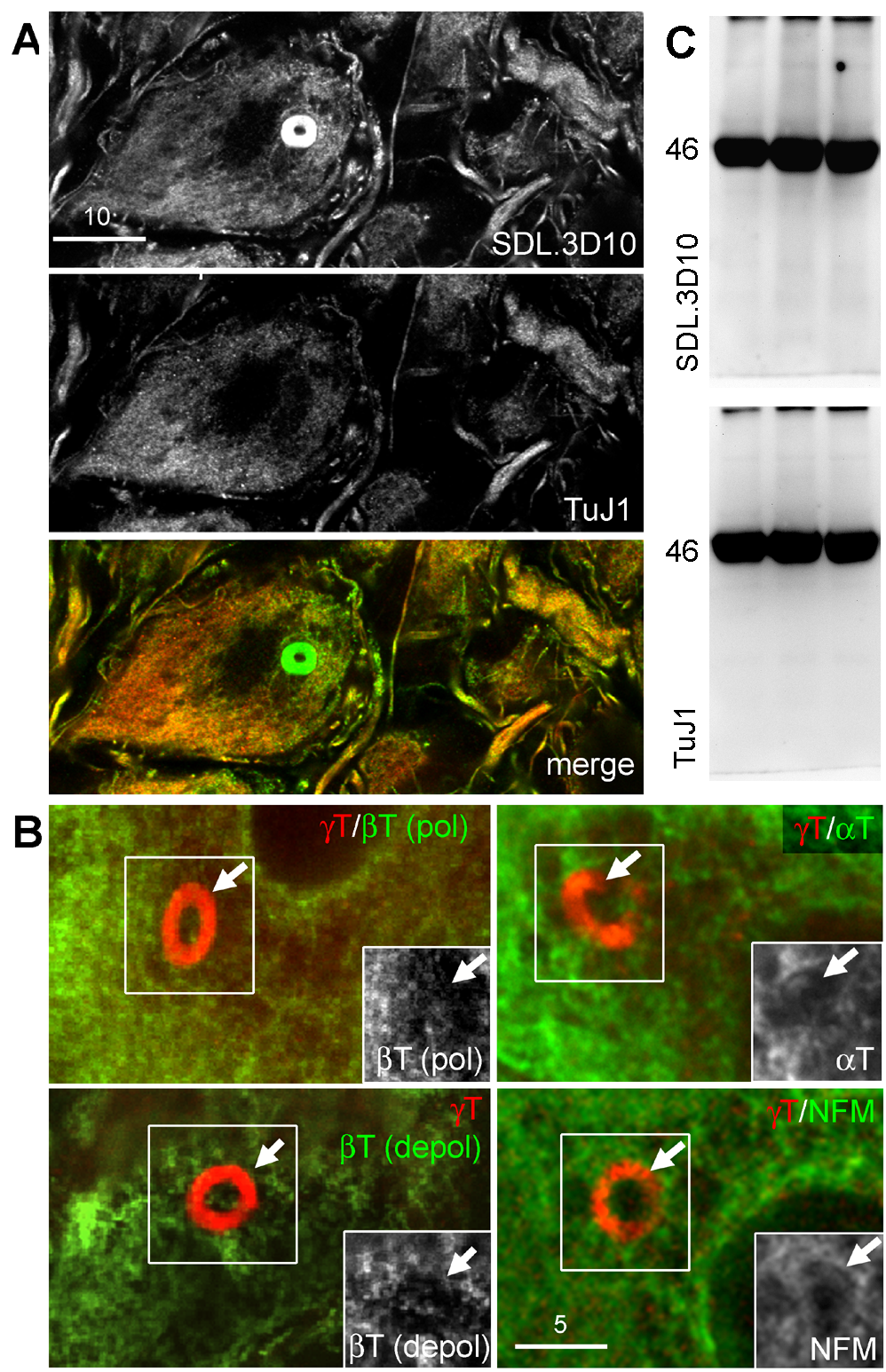

Supplement: Figure S1 — Cytoskeletal antigenicity of the loukoumasome. A, The SDL.3D10 monoclonal antibody, but not the TuJ1 rabbit polyclonal, recognizes the loukoumasome. The two antibodies otherwise recognize identical structures. B, Loukoumasomes do not contain either polymerized (βT pol), or de-polymerized (βT depol) β-tubulins. γ-tubulin-positive (γT) loukoumasomes are weakly-positive for α tubulin subunits (αT) and medium-weight neurofilament (NFM). All images: single confocal slices. C, Western blot of pelvic ganglion tissue probed for TuJ1, stripped and re-probed for SDL.3D10. In both cases a single, prominent band is visible at ∼46 kDa. Scale bar units: μm. (4.64 MB TIF) [file pone.0010872.s001.tif]
